# Environmental health recommendations for Multidrug-Resistant Tuberculosis in low- and middle-income countries: a systematic review

**DOI:** 10.1186/s12889-026-26503-4

**Published:** 2026-02-17

**Authors:** Amy Nel, Mary E. Miller, Hanna-Andrea Rother

**Affiliations:** Division of Environmental Health, Department of Public Health, University of Cape Town, Observatory, Cape Town, 7925 South Africa

**Keywords:** Multi drug-resistant, Tuberculosis, Environmental health, Outcomes, Transmission, Systematic review

## Abstract

**Background:**

The impact of environmental health risk factors on the optimization of health in MDR-TB infected individuals, as well as on the prevention of transmission to household contacts, is not well documented. Establishing the recommendations of environmental health risk factors for individuals living at home with MDR-TB will have important policy implications in LMICs, such as South Africa.

**Aims:**

The study objectives were to identify current environmental health recommendations to optimize the health of individuals infected with MDR-TB, while living at home during the completion of their treatment regimen; and to prevent transmission to household contacts by individuals infected with MDR-TB.

**Methods:**

A systematic search of electronic databases was conducted in May 2021 using a predefined search strategy. An updated search was conducted in November 2023. All primary observational studies evaluating populations *≥* 13 years of age in LMICs were included if they examined the relationship between MDR-TB and the pre-defined exposures and outcomes.

**Results:**

The results of the systematic review highlighted an important potential relationship between environmental risk factors and optimising the health of individuals infected with MDR-TB. It also emphasized the role that promoting good environmental health practices may play in preventing the transmission to household contacts and should be incorporated into local health system strategies and global policy.

**Trial registration:**

The protocol of this review was submitted and published on the PROSPERO register (Protocol registration number: CRD42021255946).

**Supplementary Information:**

The online version contains supplementary material available at 10.1186/s12889-026-26503-4.

## Background

Tuberculosis remains a prominent cause of death, particularly in low- and middle-income countries (LMICs) [1]. Despite some success in the management and prevention of Tuberculosis (TB), Multidrug-Resistant Tuberculosis (MDR-TB) may potentially compromise these endeavours as it remains significantly under addressed and has the potential to become the most dominant form of TB in low- and middle-income countries [2]. 

In South Africa, TB contributes to the quadruple burden of disease as a result of the existing significant presence of communicable and non-communicable diseases. Communicable diseases include Human immunodeficiency virus (HIV) and TB while non-communicable diseases include cardiovascular diseases, cancer and diabetes [3]. MDR-TB occurs when the organism Mycobacterium Tuberculosis shows resistance to two dominant drugs in TB treatment - Isoniazid and Rifampin. If a patient with MDR-TB does not undergo adequate treatment, the mycobacterium may develop further resistance to treatment, resulting in extensively drug resistant Tuberculosis (XDR-TB). XDR-TB is characterised by Isoniazid and Rifampin resistance, as well as resistance to additional agents in the class of fluoroquinolone medications and one or more of the second-line drugs, including Amikacin, Kanamycin, or Capreomycin [4, 5]. The current focus on the treatment and prevention of MDR-TB is mostly through new treatment regimens, treatment agents and methods for early diagnosis. However, a multi-pronged approach is desperately lacking within current MDR-TB treatment goals including the underrepresentation of the environmental health influences on MDR-TB. Previous studies have reported on identified individual health risk factors for MDR-TB in adult populations (e.g., diabetes mellitus and HIV) [6, 7]. However, the overall impact of environmental health risk factors on the optimization of health of MDR-TB infected individuals, as well as the prevention of transmission to household contacts, specifically in LMICs, is not well documented.

The vulnerability of LMIC populations to the growing prevalence of MDR-TB is exacerbated by inadequate understanding of and adherence to treatment regimens, in addition to poor surveillance in TB control programmes [8, 9]. This poses a significant burden on fragile healthcare systems within LMICs. Even though MDR-TB is more likely to emanate from a primary infection, instead of acquired resistance from poor compliance, both circumstances highlight the important significance of environmental controls for MDR-TB [10]. These also present important implications for vulnerable populations previously described as at risk for MDR-TB [11–13]. Treatment programmes for MDR-TB in LMICs have been shown to be cost-effective, however, scaling up such treatment programmes requires extensive resources for TB surveillance and prevention that addresses the role of environmental determinants of health and the underlying social determinants [14]. Therefore, the relationship between environmental health recommendations in the prevention of transmission to household contacts and the optimization of health for individuals living at home with MDR-TB, has important policy implications, especially in LMICs. This involves incorporating environmental health risk factors in current efforts towards achieving Sustainable Development Goals (SDGs) and TB related targets [15, 16]. Currently, these include achieving a 90% reduction in TB incidence rate and a 95% reduction in deaths due to TB by 2035, as well as ending the TB epidemic by 2030 (SDG 3) [15, 16]. 

Current local prevention and treatment efforts are targeted at: reducing transmission, improving infection control mechanisms, incorporating drug susceptibility testing for fast TB diagnosis, prompt initiation of appropriate treatment, contact tracing among vulnerable groups, and decreasing disease progression through preventative therapy. The importance of addressing environmental health factors within these strategies is not well represented in current local policies and practices [17]. Effective environmental health recommendations in the prevention of transmission of MDR-TB to household contacts, and the optimization of health for individuals living at home with MDR-TB while on treatment has not been well explored in previous research [6, 18]. Establishing guidance for environmental health risk factors for patients living at home with MDR-TB, in order to identify and develop targeted policy recommendations, will assist in decision making for LMICs tackling growing rates of MDR-TB.

Due to the gap in the literature, we embarked on a systematic review. The aim of the review was to identify the current environmental health recommendations for individuals in LMICs that are living at home while infected with MDR-TB, to support optimizing their health and prevent transmission to household contacts. Additionally, this review sought to identify current environmental health recommendations available in the published literature and to make further recommendations based on the study findings for policies and preventative efforts.

### Objectives

The study objectives were to identify current environmental health recommendations to optimize the health of individuals infected with MDR-TB, while living at home during the completion of their treatment regimen; and to prevent transmission to household contacts by individuals infected with MDR-TB. The final objective was to provide new recommendations based on the study findings for policies and standards to improve the well-being of individuals infected with MDR-TB, living at home. The recommendations in this review focused on addressing underlying risk factors and in doing so, recognizing that these risk factors do not directly translate into interventions but are critical targets for future policy and research.

## Methods

### Definitions

#### Environment

In this review, the term *environment* is used in a broad, integrated sense to reflect the realities of MDR-TB in LMICs, where physical conditions (e.g., overcrowding, air pollution), social determinants (e.g., poverty, poor nutrition), and certain individual behaviours (e.g., alcohol or substance use) interact, potentially affecting the overall environmental health risk. Although behaviours such as alcohol and substance use are not exclusively environmental exposures, they can intensify vulnerability to environmental risks and influence treatment engagement and outcomes. This integrated definition is therefore used to capture the interconnected physical, social, and individual factors that may collectively constitute the environmental context of individuals affected by MDR-TB [11–13].

#### Optimization of health

Although the definition of optimization of health regarding MDR-TB is broad, for the purposes of this review, it included quantification of the following indicators using standardized outcome measurements:

Treatment outcomes (cure, defaulted (incomplete) treatment, death), health status (Body Mass Index (BMI) estimates (kg/m^2^), cardiovascular endurance through walking, functional assessments of activities of daily living, positive/negative sputum cultures; or other relevant and appropriate standardised measures identified in the literature.

#### Transmission to household contacts

Assessment of household contacts and their TB status included a positive GeneXpert MTB/RIF assay of direct household contacts with diagnosed MDR-TB. Direct household contacts include any individual residing within the same residence as the index case for more than one day a week [19]. Household contacts may include children under the age of 13 in addition to the inclusion of adults as later described.

### Eligibility criteria

The systematic review was conducted in accordance with the Preferred Reporting Items for Systematic Reviews and Meta-Analyses (PRISMA) guidelines [20]. The protocol of this review was submitted and published on the PROSPERO register (Protocol registration number: CRD42021255946). All primary observational studies evaluating populations *≥* 13 years of age in LMICs were included if they examined the relationship between MDR-TB and the pre-defined exposures and outcomes. Populations from adolescents (*≥* 13 years of age) were included due to the varying definition of appropriate adult ages within the literature. Therefore, studies done on children (< 13 years) were excluded, except if evaluating child household contacts of MDR-TB cases. This approach was adopted to ensure consistency in population definitions. Screening was conducted by one independent researcher (AN) and reviewed by a second independent researcher (MEM). Articles were included from 2000 through the study year (2021), to ensure that only recent and current research was included. Only articles for which the full text version was available were included. Studies were excluded if they evaluated drug-resistant TB other than MDR-TB/XDR-TB, evaluated the environmental health recommendation categories not relevant to the systematic review or used qualitative methods. To ensure that the findings and recommendations of this review remained generalisable to the general population, studies of populations with another specific pre-existing disease were excluded (e.g., cancer). Duplicate publications of identical material, narrative reviews, and any other publications with absent primary data, or a clear method description were also excluded.

### Literature search

A systematic search of electronic databases was conducted in May 2021 using a predefined search strategy. The search strategy was adapted for each database and followed PRISMA guidelines. An example of search terms has been provided in Table 1.

**Table 1:** Example of search strategy for environmental health recommendations to optimize health and prevent transmission to household contact of Multidrug resistant TB patients

Pubmed search string	(Tuberculosis, Multidrug-Resistant [MeSH] OR Extensively drug resistant TB [MeSH] OR Multidrug-Resistant Tuberculosis[Text Word] OR Multidrug-Resistant TB[Text Word] OR Drug-Resistant Tuberculosis[Text Word] OR Drug-Resistant TB[Text Word] OR MDR Tuberculosis[Text Word] OR MDR TB[Text Word] OR Extensively drug resistant Tuberculosis[Text Word] OR XDR TB[Text Word]) AND (Risk Factors [MeSH] OR Socioeconomic Factors [MeSH] OR Malnutrition [MeSH] OR Smoking [MeSH] OR Alcoholism [MeSH] OR Alcohol Drinking [MeSH] OR Population Dynamics [MeSH] OR Indoor air pollution [MeSH] OR Outdoor air pollution [MeSH] OR Overcrowding[Text Word] OR ventilation[Text Word] OR diet[Text Word] OR malnutrition[Text Word] OR malnourished[Text Word] OR undernourishment[Text Word] OR starvation[Text Word] OR smoking[Text Word] OR poverty[Text Word] OR socioeconomic[Text Word] OR alcohol[Text Word] OR alcoholism[Text Word] OR urbanization[Text Word] OR migration[Text Word]) AND (Infectious Disease Transmission [MeSH] OR Optimize health [MeSH] OR Transmission[Text Word] OR infectiousness[Text Word] OR containment[Text Word] OR prevention[Text Word] OR optimal health[Text Word])

A waiver of ethical approval was granted by the Departmental Research Committee of the School of Public Health and Family Medicine, University of Cape Town (UCT). Databases that were searched included: Ebscohost (collections included Africa-wide information, Cinahl, Medline, health source nursing edition); Web Science (included core collection and Scielo); Scopus and Pubmed. After the removal of duplicates, the articles were screened via their title and abstract for eligibility for inclusion into the final sample. If the inclusion criteria were met, the full text of the article was retrieved.

### Description of study selection

A review of the final sample of potentially eligible articles was conducted by evaluating the full texts, with only those available in English included in the final sample. The screening and full-text review were performed independently by one reviewer with oversight from another independent reviewer. Following the identification of relevant articles, a manual review of reference lists and grey literature was conducted to identify any additional applicable studies meeting the inclusion criteria. An updated search, following the predefined search strategy from June 2021 to 2023, was also carried out, yielding no further articles that met the inclusion criteria. Data extraction and charting of results were performed by one independent reviewer with oversight and verification by the second reviewer using a data extraction form specifically designed for this review. Any discrepancies during screening, extraction, or charting were resolved through discussion or consultation with a third reviewer to ensure accuracy and consistency.

**Fig. 1:**
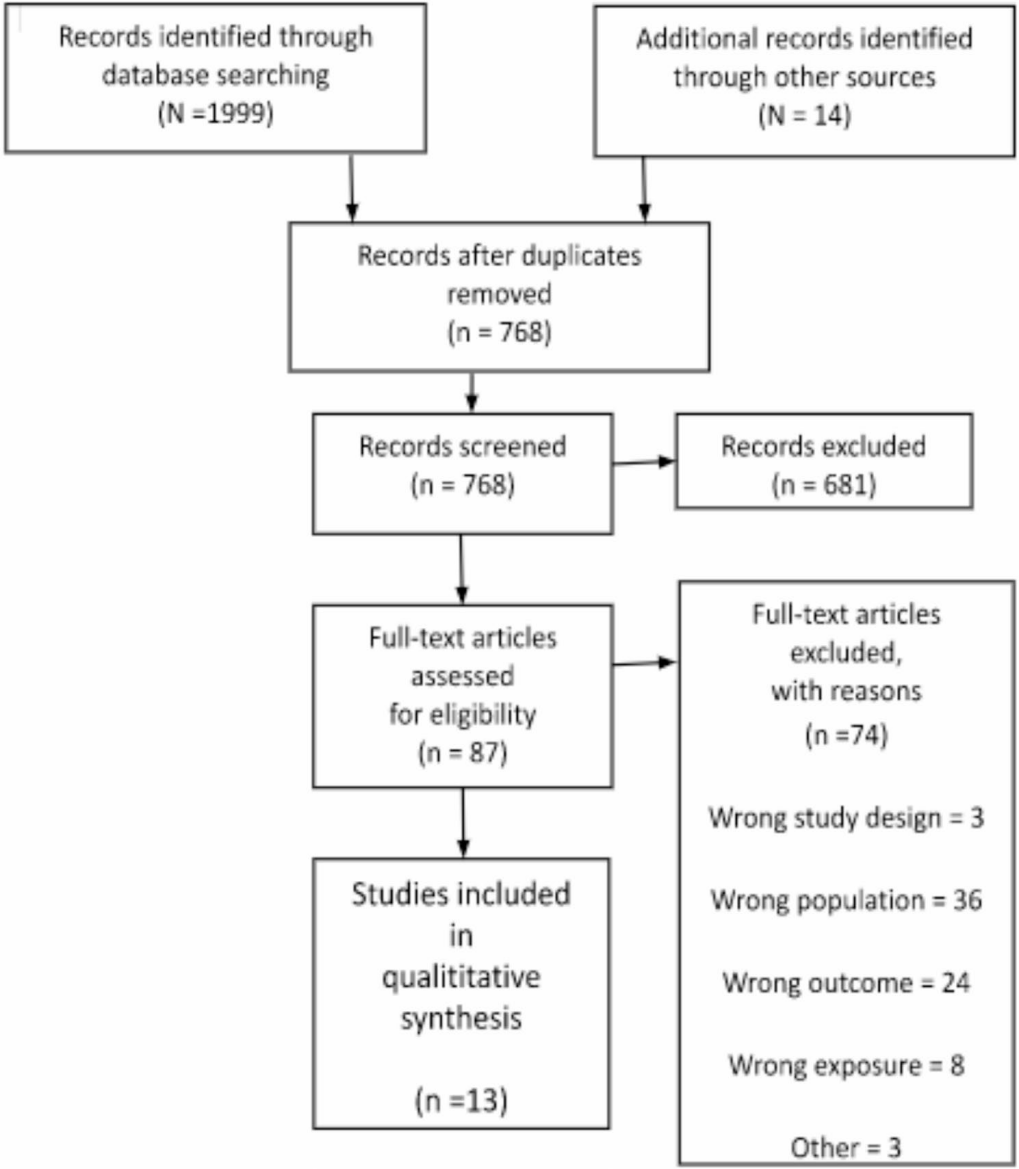
PRISMA Flow diagram

The study selection results are presented in the PRISMA flow diagram (Fig. 1). After the initial screening of databases, a total of 1999 articles were found with an additional 14 articles included from manual searching of additional sources as described in the protocol (i.e., screening of reference lists, grey literature search and Google scholar). Initial screening of 768 articles without duplicates was conducted, of which 681 were excluded. Of the remaining 87 sample articles for full text review, 74 articles were excluded and 13 met the inclusion criteria for the final sample. A high number of studies were excluded during full-text screening primarily due to a mismatch with the target population. These exclusions were necessary to maintain the integrity and focus of the review, as including studies on general TB populations or unrelated environmental exposures would have reduced the specificity and relevance of the findings. The final sample, therefore, consisted of 13 articles which were included in the narrative synthesis.

### Data collection process

The final included sample was then reviewed for data extraction. The data extraction process was done by one reviewer (AN) and final extracted data was reviewed by two independent researchers (MEM and HAR). Articles that were included in the initial selection for full text review, but were identified as ineligible for the study, have been listed with reasons for exclusion within the PRISMA flow diagram (Fig. 1).

### Quality assessment

Quality assessment of the final included sample was assessed using pre-specified standardized tools namely the Joanna Briggs Institute (JBI) Critical Appraisal Checklist for cohort studies, the JBI Critical Appraisal Checklist for case-control studies and the JBI Critical Appraisal Checklist for analytical cross-sectional studies [21]. This process was conducted by one independent reviewer (AN) and the final assessment was reviewed by two additional independent reviewers (MEM and HAR). Most of the included studies were cohort studies (*n* = 11). All cohort studies met ≥ 7 criteria on the JBI checklist, with only one study meeting all criteria (11/11 score). The areas of concern for cohort studies were the identification of confounders and strategies to deal with confounders of which only three articles reported doing this. Other under-performing criteria were follow-up completion, reasons for loss to follow up, and strategies to accommodate any missing data. One cross-sectional study was included in the review, which met most of the quality assessment criteria (6/8), however, potential confounders and strategies used to address these confounders were unclear within this article. Finally, one case control study was included in the review. This study met 8 out of 10 criteria for the quality assessment, although again, it was not clear if potential confounders were identified or if strategies were utilised to address confounders. These quality assessments were used to guide the interpretation of the evidence in the synthesis and to indicate where the conclusions should be interpreted with greater caution.

## Results

### Description of included studies

 The extracted study characteristics and main findings of the included articles are presented in Table 2. The final sample included eleven cohort designs, one cross sectional study and one case control study. The variability in methodology contributed to the heterogeneity of the data, precluding any attempts at a meta-analysis. The final sample was grouped according to the two outcomes this review sought to analyze. Ten studies evaluated the optimization of health outcomes and three evaluated disease transmission to household contacts as an outcome. Specifically, most studies assessing the optimization of health, evaluated treatment outcomes and mortality in MDR-TB patients. The final sample evaluated various environmental health exposures including: smoking [22–24], nutrition [19, 22], – [25] household condition [19, 26–28], alcohol or other substance use [23, 26, 29–32], and migration [31]. The search yielded no eligible articles related to indoor and outdoor air pollution or urbanisation. The final sample of included studies was from various LMICs globally and evaluated MDR-TB and XDR-TB according to predefined definitions, which were appropriate for inclusion into the review sample. Additionally, all studies were undertaken in regions which have a significant TB burden according to the WHO [33].

**Table 2:** Characteristics and findings of included studies

Author	Study design	Country	Participants	Sample size	Relevant environmental health exposure	Outcomes	Main findings^1^
Transmission to household contacts							
Grandjean et al., 2011 [29]	Retrospective cohort	Peru	Household contacts of individuals with MDR-TB	2112 contacts within 358 households	Household factors	Household contacts with TB disease	• Increased risk of TB observed in male contact [HR^#^2.82,p-value = 0.03] • No significant differences found between crowding and transmission.
Golla et al., 2017 [19]	Cross sectional	South Africa	Child household contacts older than 5 years	545 patients	Smoking, household factors, nutrition	Household contacts with TB infection and TB disease.	• Children who had TB infection were more likely to have been in contact with an adult source case [aOR*2.05 (1.34–3.12.34.12)] • The risk for TB disease in children who were underweight for their age was non-significant [OR** 1.04 (0.56–1.92.56.92)] • Household tobacco smoke exposure was a non-significant risk factor for TB disease in children [aOR 1.22 (0.76–1.94.76.94)]
Shadrach et al., 2021 [25]	Prospective cohort	India	Household contacts of individuals with MDR-TB	271 patients	Nutrition	TB occurrence in household contacts	• Being less than 18 years of age was found to be a non-significant risk factor for TB [OR 0.72 (0.43–1.82.43.82)] • Both male sex [OR 2.3 (1.35–3.94.35.94)] as well as malnourishment [OR 1.89 (1.13–3.16.13.16)] were identified as risk factors for TB
Optimisation of health							
Bei et al., 2018 [22]	Retrospective cohort	China	Patients aged 19–99 years with XDR-TB	72 participants	Smoking, nutrition	Mortality	• Smoking [aHR## 4.67 (1.66–13.16.66.16)] and BMI < 18.5kg/m2 [aHR 4.5 (1.3–15.7.3.7)] were significant predictors of mortality.
Chung Delgado et al., 2015 [23]	Retrospective cohort	Peru	Patients aged 18 years and above with MDR-TB	1,242 patients	Alcohol, smoking, nutrition	Mortality rates; mortality associated risk factors during treatment of MDR-TB	• Non-significant factors associated with mortality: ◦ Smoking: [HR 0.40 (0.12–1.29.12.29)] ◦ Alcohol: [HR 0.51 (0.21–1.20.21.20)] ◦ Body mass index: ◦ underweight [HR 1.84 (0.97–3.50.97.50)] ◦ Overweight/obese [HR 1.14 (0.46–2.84.46.84)]
Cohen et al., 2011[28]	Retrospective cohort	Peru	MDR-TB isolates from patients in eligible households	102 households with 232 participants	Household factors	MDR-TB reinfection	• Household size, density and quality were all unrelated to multiple introduction events.
Franke et al., 2008 [26]	Retrospective cohort	Peru	All patients with laboratory confirmed MDR TB	671 patients	Substance use, housing, nutrition	Cure, treatment completion, treatment failure, mortality	• Substance use (unspecified) was a dominant risk factor for treatment default [aHR 2.96 (1.56–5.62.56.62)] • This finding was observed primarily among men. • Substandard housing conditions predicted treatment default [aHR1.83 (1.07–3.11.07.11)]
Holtz et al., 2006 [31]	Case control	South Africa	Patients aged 18 years and older diagnosed with MDR-TB	269 confirmed cases and 401 controls	Smoking, other substance use, migration	Mortality among MDR-TB treatment defaulters	• Default of treatment was associated with the following: ◦ Smoking marijuana or mandrax during treatment [aOR 17.9 (4.7–68.5 ◦ Having a place of birth outside of South Africa [OR 5.9 (1.1–32.8.1.8)] ◦ Having a change in residence during treatment period [aOR 3.2 (1.4–7.6.4.6)]
Kendall et al., 2013 [32]	Retrospective cohort	South Africa	All patients (age > 15 years) starting MDR-TB treatment	225 patients	Alcohol, substance use. household factors	Cure or treatment completion; treatment failure, mortality	• Predictors of default during outpatient treatment: ◦ Alcohol [aHR 2.11 (1.11–4.02.11.02)] ◦ Drug use [aHR 2.02 (1.04- 3.95)] • Having water, toilet and electricity in dwelling was not associated with default/death or treatment failure [HR 0.63 (0.33–1.20.33.20)] • Formal dwelling was associated with a lower rate of default [HR 0.38 (0.19–0.78.19.78)]
Lu et al., 2017 [26]	Prospective cohort	China	Age group not specified. Median age = 51 years	160 patients	Smoking, alcohol, nutrition	Sputum conversion	• Risk factors affecting culture conversion: ◦ Smoking [aHR 0.44 (0.23–0.83.23.83)] ◦ Alcohol [aHR 0.41 (0.21–0.81.21.81)]
Shean et al., 2008 [30]	Retrospective cohort	South Africa	Patients aged ⩾15 years initiating treatment for pulmonary MDR-TB	747 patients	Alcohol use	Cure and completion were regarded as successful outcomes, poor outcomes included death, default of treatment and failure of treatment.	• Current heavy alcohol use was associated with poor treatment outcomes [RR† 1.3 (1.02–1.7.02.7)]
Qazi et al., 2011 [24]	Retrospective cohort	Pakistan	MDR-TB patients confirmed on culture	85 participants	Nutrition, smoking	Culture conversion	• Current smokers had a higher chance of culture conversion compared to individuals who never smoked [HR 0,08 (0,01-0,49)]
Savioli et al., 2019 [28]	Cohort	Brazil	Patients 18 years or older, with confirmed MDR-TB	190 participants	Nutrition	Initial outcome events (abandonment, failure, cure and death)	• Patients who had no comorbidities had a higher chance of cure specifically after 18-month treatment period [OR 3.37 (1.41–8.09.41.09)] • An increased initial weight did not indicate a significant chance of cure after 18-month treatment period [OR 1.04 (0.99–1.08.99.08)]

^1^Includes reported effect measure and confidence interval/p-value

* *aOR* adjusted odds ratio ** *OR* odds ratio # *HR * hazard ratio ## *aHR * adjusted hazard ratio † *RR* risk ratio

### Transmission to household contacts

A study examining transmission to household contacts found a non-significant relationship between child household contacts of individuals with MDR-TB who had confirmed TB disease and being underweight for their age (using BMI as an estimate) [19]. The relationship between exposure to household tobacco smoke and confirmed TB disease in child household contacts of individuals with MDR-TB was also not significant [19]. Additionally, overcrowding was not a significant predictor of TB disease among contacts of household members with MDR-TB [27]. Only one study evaluated nutrition, concluding that malnutrition in household contacts of MDR-TB positive household members, posed an increased risk of TB disease [34]. 

### Optimisation of health

A large proportion of articles evaluated outcomes relating to optimization of health through mortality-related outcomes, default of treatment, or completion of treatment outcomes [22, 23, 26, 30, 31]. Two studies utilised sputum conversion as an outcome measure [24, 29]. Smoking was identified as a significant predictor of mortality in one study [22], however, produced a contradictory result in another [23]. Smoking was also found to have a significant adverse effect on sputum culture conversion in MDR-TB patients receiving treatment [24, 29]. Substance use (i.e., marijuana or mandrax or unspecified) was identified as a significant risk factor for defaulting on treatment [26, 31, 32]. The use of alcohol was commonly reported as an indicator for poor treatment outcomes [30] and defaulting on treatment [32]. The use of alcohol was also found to be a significant factor negatively affecting sputum culture conversion within individuals positive for MDR-TB [29]. However, one study reported a non-significant relationship between alcohol consumption and mortality in individuals with MDR-TB [23]. 

Pertaining to household conditions, an association was found between substandard housing conditions and treatment default, even though socioeconomic support was provided to vulnerable patients [26]. Conversely, formal housing had a lower rate for treatment default [32]. Only one study examined the relationship between having water, a toilet, and electricity in the dwelling and default rate, however, no significant relationship was found [32]. Another study found that the household size and density as well as the quality of the household structure were unrelated to multiple MDR-TB introduction events [28]. The role of migration was not explored in depth, although one study reported that change of residential address was associated with treatment default [31]. 

In terms of nutrition, some studies found that BMI estimates less than 18.5 kg/m^2^ (underweight for age) were significantly associated with increased mortality [22]. However, another found that the there was no significant association present for underweight/overweight/obese BMI estimates [23]. In addition, patients with MDR-TB who had a higher initial weight prior to commencement of treatment did not have a significant chance of cure [2]. 

### Vulnerable populations

Gender differences were reported in some of the included studies. Two studies reported that males are more at risk for TB disease than females [27, 34]. Additionally, male household contacts were found to have a higher incidence of TB compared to female contacts [27]. Worth noting is that most studies included a population that was mostly represented by the male gender (> 50%) [22, 23, 25, 28–30]. A reduction in the relationship observed between female sex and death occurring after default on treatment, if certain confounders were accounted for, such as, inadequate bacteriologic response, having been on an individualized treatment programme for less than a year, level of education, and history of psychiatric disorder. This suggests that the aforementioned factors possibly mediate the relationship existing between death after treatment default and female sex [26]. 

The included studies also highlighted the impact of HIV on the optimisation of health for individuals who are living at home with MDR-TB. Within the included studies, HIV was associated with a greater incidence of death in those with MDR-TB [23]. Conversely, some studies did not find an association for defaulting treatment in HIV-positive individuals [26, 32] nor a significant association between BMI and culture conversion in HIV-positive individuals [24, 29]. Additionally, some studies did not record HIV status within their study population, which may have had an impact on the relationship between environmental risk factors and health optimisation or transmission [29, 31, 32]. 

Children were suggested to be particularly vulnerable if living in households with an individual who has MDR-TB. Although only three of the included studies reported on transmission to household contacts, it was found that children ≥ 2 years of age who developed TB infection were more likely to have been in contact with an adult TB source case in comparison to children who were TB-exposed but uninfected [19]. Interestingly, within the same study, children underweight for their age did not have a significant risk of TB disease nor were they more likely to have been exposed to household tobacco smoke [19]. 

## Discussion

This study highlights the role of environmental health risk factors in MDR-TB transmission prevention efforts, specifically in LMICs. Several environmental health factors including smoking, household condition, nutrition, alcohol, other substance use, urbanisation and migration were reviewed.

### Smoking

Although not a new concept, the findings reiterate the need for improved legislation and re-evaluation of the externalities suffered by vulnerable populations who are exposed to individuals who smoke [35]. This is especially important in LMICs, where legislation surrounding tobacco exposure may not be as stringent or protective as required for vulnerable populations. To address the environmental health impact of smoking, efforts toward increased outreach and education should be utilised. This includes individual education provided by community health workers, education in a clinical setting, and health education campaigns that bring attention to the community regarding the health impacts of smoking and MDR-TB [16]. 

### Substance and alcohol use

Although substance and alcohol use are not specific environmental health risk factors, they are both significant social issues, particularly in LMICs, that may have considerable influence in predisposing vulnerable individuals, even more so to environmental health risk factors. Through awareness of the impact of these factors on MDR-TB outcomes and transmission, any local or global intervention to combat TB and MDR-TB should prioritise social support in such population groups. This also extends to stricter legislation and education on the impact that other substance use, and alcohol have in the context of MDR-TB [36]. 

### Nutrition

Nutrition appears to have a significant impact on individuals with MDR-TB, as it is thought that poor nutrition increases the chance of TB infection and negatively impacts the immune response, making infected individuals more vulnerable to poor outcomes [37]. Strategies to address this include additional nutritional supplements, feeding programmes and improved access to dieticians and community health workers. Such programmes and interventions require intense scale-up and investment from larger stakeholders and communities, ensuring successful and sustainable programmes [37]. 

### Physical housing

Evaluation of the impact of housing was limited to overcrowding and personal habits of individuals (i.e., tobacco exposure), rather than physical attributes of households. In addition, different types of cooking sources, which have been described in previous studies evaluating general TB risk, were not explored within the included studies as the available studies did not meet the exclusion criteria [38]. This is a field that should be evaluated in further research. Furthermore, the link between MDR-TB outcomes, transmission incidence, and air pollution were not described. Thus, it is unclear what the relationship is between MDR-TB transmission and households using different fuel sources, or households that are close to outdoor air pollution sources. The evaluation of housing conditions and MDR-TB also identified vulnerable populations. Children living in households with MDR-TB-positive individuals, may be particularly vulnerable to transmission in poor housing structures, due to their known vulnerability to environmental exposures [39]. 

### Gender vulnerabilities

As mentioned, previous research has reported gender vulnerabilities for contracting TB, where males were identified as having a higher risk of acquiring TB particularly, in LMICs [6, 40]. Conversely, women appear to be exposed to greater amounts of indoor air pollution due to using solid fuel for cooking purposes and as a result, possibly placing them at higher risk [38]. This controversy and lack of gender-disaggregated data warrants further investigation as the consequences thereof are important in the achievement of SDGs grounded in gender equality (SDG 5), as well as for prioritization of vulnerable gender groups in national health strategies targeting MDR-TB. This is a clear gap in current research which warrants further investigation.

### Migration and urbanisation

This review was unable to draw any firm conclusions regarding the role of migration and urbanisation due to limited evidence available. Both are complex themes which are under considerable socioeconomic and environmental influence. Thus, a conclusion regarding their influence in optimising health outcomes as well as transmission prevention requires further investigation. Urbanisation may have significant influence on an individual’s access to housing, type of housing structure, and household and community crowding. It may also increase migration for job opportunities, health facilities, as well as to improve nutritional resources [16]. These complex themes require further research to fully understand their influence in MDR-TB prevention.

### Policy implications

This review highlights potential considerations for policy and decision making in LMICs such as South Africa. Firstly, the role of environmental health may warrant further exploration regarding how it could be incorporated into actionable objectives to attain local as well as global policy goals. The SDGs are examples whereby the role of the environment and its impact on health appear to be increasingly recognised in addressing the management of MDR-TB [15]. Secondly, local and national health guidelines developed for the prevention and management of MDR-TB could consider recognising and including relevant steps in promoting or providing environments that are conducive to health. This may contribute to more comprehensive health decisions, with the environment as a key consideration in the prioritisation of an individual’s health and fulfillment of basic human rights. Thirdly, identified vulnerable populations (e.g., women, children and HIV-positive populations) could be considered for prioritisation in local and global policies addressing the management and prevention of MDR-TB, as this may help address the injustice and inequality that exists for such groups. Finally, the role of stakeholder engagement and financial prioritisation from governments might represent an important future focus in addressing environmental health issues in the context of MDR-TB management and prevention.

There are limitations to this study which can be attributable to the nature of the primary data obtained in the review process. Due to the heterogeneity of the primary data, as well as results of the quality appraisal, interpreting the results should be done with caution. The quality of studies included varied significantly. In addition, the results may have been influenced by unidentified confounding variables or by inappropriate strategies to address missing data caused by loss to follow up. Most studies included data which relied on self-reporting, which could be significantly impacted by recall bias. Some studies also relied on a convenience sampling method; therefore, the results may be prone to selection bias, potentially impacting the generalisability of the results. In addition to the complexity of the subject matter, search terms may have oversimplified major themes such as urbanization and migration. This may have resulted in the underrepresentation of the role that poverty and the effect thereof may have in the outcomes of MDR-TB treatment. Thus, the complexity of these themes should further be critically analysed in future work. Due to the limited quantity of available data pertaining to the subject, interpretation of the results cannot be done with full confidence unless further data becomes available. The extremely small pool of available evidence limits the breadth of conclusions that can be drawn and raises concerns about the representativeness of the findings. Additionally, the potential for publication bias may further skew the available evidence base. However, the results do provide a platform for this gap in research to be highlighted and provides motivation for future research. This is particularly important in understanding the role of air pollution, a prominent environmental risk factor, in MDR-TB treatment and prevention. This review used a single reviewer for study screening, full-text assessment and data extraction. Although data extraction was subsequently checked by two team members, the absence of dual independent screening may have introduced selection or extraction bias. This approach reflects pragmatic constraints, and the verification processes used partially mitigate this limitation. Nevertheless, the potential for missed studies or reviewer subjectivity must be acknowledged when interpreting the findings. Finally, limiting studies in this review to those published in English may also have neglected to identify potentially relevant research.

## Conclusion

The results of this review highlight the possible relationship between environmental risk factors and the health of individuals with MDR-TB, as well as the role that environmental health could potentially play in preventing the transmission of MDR-TB to household contacts. While the available evidence is limited, heterogeneous, and sometimes inconsistent, these findings suggest that environmental health may be an important, yet underrepresented, component in MDR-TB prevention and management strategies. Local and global policies, including WHO targets and SDGs, might consider integrating environmental health more explicitly into strategies to provide actionable goals in improving the health of individuals with MDR-TB, as well as to prevent transmission to exposed household contacts. However, given the variability in study quality and the methodological limitations identified, these suggestions should be interpreted cautiously. Further high-quality, context-specific research is essential to better understand the extent and mechanisms through which environmental factors influence MDR-TB outcomes and the optimisation of health. A stronger evidence base would help inform the development of more targeted, effective, and equitable policy responses.

## Supplementary Information

Supplementary Material 1.

Supplementary Material 2.

Supplementary Material 3.

## Data Availability

No datasets were generated or analysed during the current study.

## Declarations

This article was part of a partial requirement of a Master of Public Health (MPH) at the University of Cape Town.

## Abbreviations

BMI: Body Mass Index
DRC: Departmental Research Committee
HIV: Human immunodeficiency virus
JBI: Joanna Briggs Institute
LMICs: Low- and middle-income countries
MDR-TB: Multidrug-Resistant Tuberculosis
SDGs: Sustainable Development Goals
TB: Tuberculosis
UCT: University of Cape Town
XDR-TB: Extensively drug-resistant TB
PRISMA: Preferred Reporting Items for Systematic Reviews and Meta-Analyses
MPH: Master of Public Health
