# Supplementary material for: Environmental health recommendations for Multidrug-Resistant Tuberculosis in low- and middle-income countries: a systematic review
Source: BMC Public Health. 2026 Feb 17;26:974. doi: 10.1186/s12889-026-26503-4 (PMC13014885; doi:10.1186/s12889-026-26503-4)
Supplement: Supplementary file 1 — Supplementary Material 1. [file 12889_2026_26503_MOESM1_ESM.pdf]

Supplementary document B

Appendix 2: Quality appraisal of final included sample

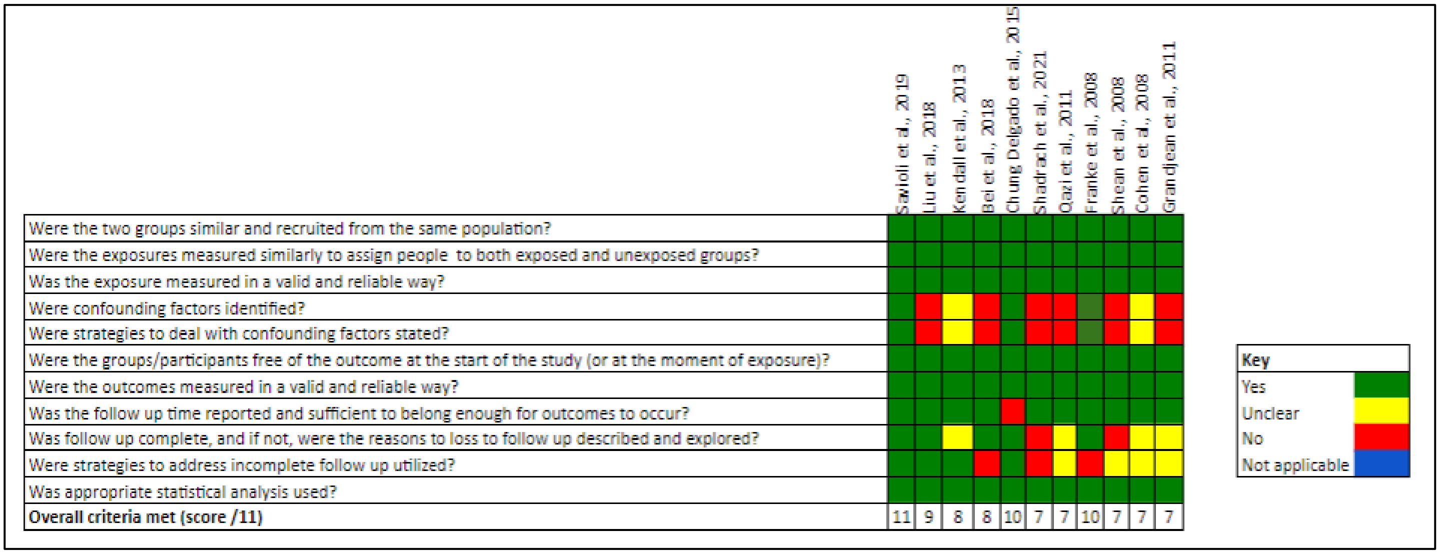

Fig. 1 Quality appraisal for cohort studies

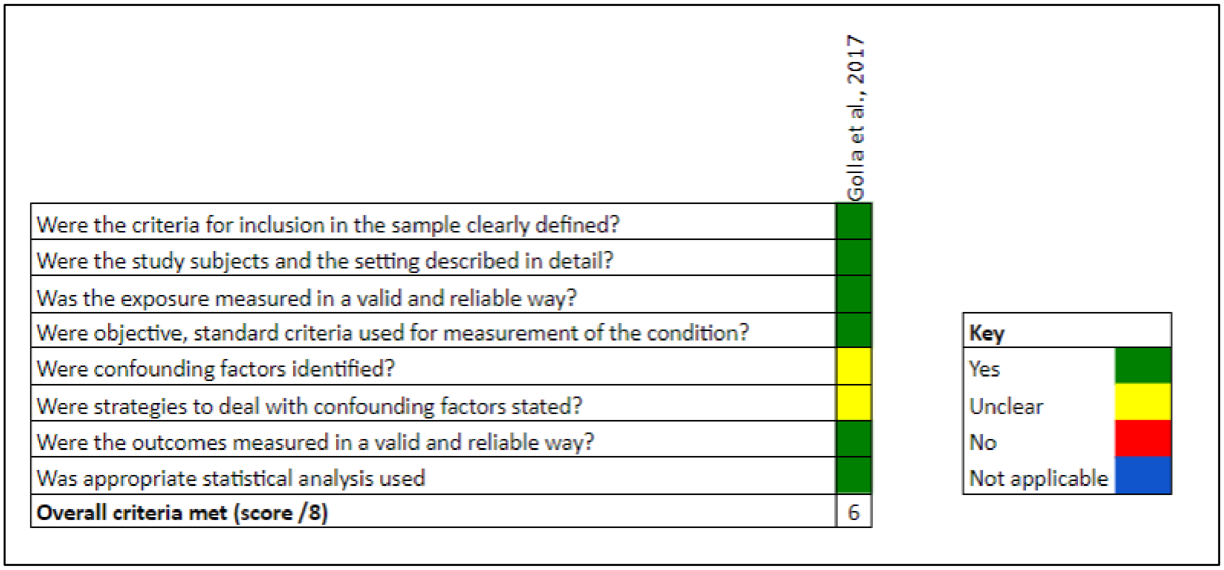

Fig. 2 Quality appraisal for cross sectional study

	Holtz et al., 2006
Were the groups comparable other than the presence of disease in cases or the absence of disease in controls?	
Were cases and controls matched appropriately?	
Were the same criteria used for identification of cases and controls?	
Was exposure measured in a standard, valid and reliable way?	
Was exposure measured in the same way for cases and controls?	
Were confounding factors identified?	
Were strategies to deal with confounding factors stated?	
Were outcomes assessed in a standard, valid and reliable way for cases and controls?	
Was the exposure period of interest long enough to be meaningful?	
Was appropriate statistical analysis used	
**Overall criteria met (score /10)**	8

**Key**	
Yes	
Unclear	
No	
Not applicable	

Fig. 3 Quality appraisal for case control study
