# Supplementary material for: Environmental health recommendations for Multidrug-Resistant Tuberculosis in low- and middle-income countries: a systematic review
Source: BMC Public Health. 2026 Feb 17;26:974. doi: 10.1186/s12889-026-26503-4 (PMC13014885; doi:10.1186/s12889-026-26503-4)
Supplement: Supplementary file 2 — Supplementary Material 2. [file 12889_2026_26503_MOESM2_ESM.pdf]

<u>Supplementary document A - Search strategy</u>

**Database**	**Search strings used**	**Date of search**	**Records identified**
Ebscohost (included Africa-wide, CINAHL, Medline, Health source nursing edition)	"Multidrug-Resistant Tuberculosis" OR "Multidrug-Resistant TB" OR "Drug-Resistant Tuberculosis" OR "Drug-Resistant TB" OR "MDR Tuberculosis" OR "MDR TB" OR "Extensively drug resistant Tuberculosis" OR "XDR TB"<br>AND<br>Overcrowding OR ventilation OR diet OR malnutrition OR malnourished OR undernourishment OR starvation OR smoking OR poverty OR socioeconomic OR alcohol OR alcoholism OR urbanization OR migration<br>AND<br>Transmission OR infectiousness OR containment OR prevention OR "optimal health"	18/05/2021	684
Web of Science (included Core collection, Medline and Scielo)	"Multidrug-Resistant Tuberculosis" OR "Multidrug-Resistant TB" OR "Drug-Resistant Tuberculosis" OR "Drug-Resistant TB" OR "MDR Tuberculosis" OR "MDR TB" OR "Extensively drug resistant Tuberculosis" OR "XDR TB"<br>AND<br>Overcrowding OR ventilation OR diet OR malnutrition OR malnourished OR undernourishment OR starvation OR smoking OR	18/05/2021	412

	poverty OR socioeconomic OR alcohol OR alcoholism OR urbanization OR migration<br>AND<br>Transmission OR infectiousness OR containment OR prevention OR “optimal health”		
Scopus	“Multidrug-Resistant Tuberculosis” OR “Multidrug-Resistant TB” OR “Drug-Resistant Tuberculosis” OR “Drug-Resistant TB” OR “MDR Tuberculosis” OR “MDR TB” OR “Extensively drug resistant Tuberculosis” OR “XDR TB”<br>AND<br>Overcrowding OR ventilation OR diet OR malnutrition OR malnourished OR undernourishment OR starvation OR smoking OR poverty OR socioeconomic OR alcohol OR alcoholism OR urbanization OR migration<br>AND<br>Transmission OR infectiousness OR containment OR prevention OR “optimal health”	18/05/2021	301
Pubmed	(Tuberculosis, Multidrug-Resistant [MeSH] OR Extensively drug resistant TB [MeSH] OR Multidrug-Resistant Tuberculosis[Text Word] OR Multidrug-Resistant TB[Text Word] OR Drug-Resistant Tuberculosis[Text Word] OR Drug-Resistant TB[Text Word] OR MDR Tuberculosis[Text Word] OR MDR TB[Text Word] OR	18/05/2021	663

	Extensively drug resistant Tuberculosis[Text Word] OR XDR TB[Text Word]) AND (Risk Factors [MeSH] OR Socioeconomic Factors [MeSH] OR Malnutrition [MeSH] OR Smoking [MeSH] OR Alcoholism [MeSH] OR Alcohol Drinking [MeSH] OR Population Dynamics [MeSH] OR Indoor air pollution [MeSH] OR Outdoor air pollution [MeSH] OR Overcrowding[Text Word] OR ventilation[Text Word] OR diet[Text Word] OR malnutrition[Text Word] OR malnourished[Text Word] OR undernourishment[Text Word] OR starvation[Text Word] OR smoking[Text Word] OR poverty[Text Word] OR socioeconomic[Text Word] OR alcohol[Text Word] OR alcoholism[Text Word] OR urbanization[Text Word] OR migration[Text Word]) AND (Infectious Disease Transmission [MeSH] OR Optimize health [MeSH] OR Transmission[Text Word] OR infectiousness[Text Word] OR containment[Text Word] OR prevention[Text Word] OR optimal health[Text Word])		
